# The Role of Glucocorticoid Receptor Signaling in Bladder Cancer Progression

**DOI:** 10.3390/cancers10120484

**Published:** 2018-12-04

**Authors:** Hiroki Ide, Satoshi Inoue, Hiroshi Miyamoto

**Affiliations:** 1 Department of Urology, Keio University School of Medicine, Tokyo 160-8582, Japan; h-ide@fc4.so-net.ne.jp; 2 Department of Urology, Tokyo Saiseikai Central Hospital, Tokyo 180-0073, Japan; 3 Department of Pathology & Laboratory Medicine, University of Rochester Medical Center, Rochester, NY 14642, USA; inosts411@gmail.com; 4 James P. Wilmot Cancer Institute, University of Rochester Medical Center, Rochester, NY 14642, USA; 5 Department of Urology, University of Rochester Medical Center, Rochester, NY 14642, USA

**Keywords:** androgen receptor, compound A, dexamethasone, estrogen receptor, glucocorticoid receptor, glucocorticoids, transactivation, transrepression, tumor progression, urothelial cancer

## Abstract

Previous preclinical studies have indicated that the activation of glucocorticoid receptor signaling results in inhibition of the growth of various types of tumors. Indeed, several glucocorticoids, such as dexamethasone and prednisone, have been prescribed for the treatment of, for example, hematological malignancies and castration-resistant prostate cancer. By contrast, the role of glucocorticoid-mediated glucocorticoid receptor signaling in the progression of bladder cancer remains far from being fully understood. Nonetheless, emerging evidence implies its unique functions in urothelial cancer cells. Moreover, the levels of glucocorticoid receptor expression have been documented to significantly associate with the prognosis of patients with bladder cancer. This review summarizes the available data suggesting the involvement of glucocorticoid-mediated glucocorticoid receptor signaling in urothelial tumor outgrowth and highlights the potential underlying molecular mechanisms. The molecules/pathways that contribute to modulating glucocorticoid receptor activity and function in bladder cancer cells are also discussed.

## 1. Introduction

Urinary bladder cancer, which is mostly a urothelial carcinoma, is one of the most frequently diagnosed neoplasms, with nearly 550,000 new cases and 200,000 deaths estimated in 2018 worldwide [1,2]. Importantly, the prognosis for bladder cancer patients remained largely unimproved, during the last few decades, in spite of considerable advances in diagnostic technologies, as well as treatment strategies [3,4]. Further research is, thus, required to better understand the molecular mechanisms responsible for the development and progression of urothelial cancer, which may subsequently provide novel targeted therapy.

Several risk factors for bladder cancer have been identified, which include tobacco use, occupational exposure to aromatic amines or polycyclic aromatic hydrocarbons, and treatment with cyclophosphamide [1,4,5,6]. In addition, the long-term systemic use of glucocorticoids has been shown to be associate with an increased risk of developing bladder cancer, presumably due to immunosuppression [7]. Meanwhile, intrinsic factors have also been documented to involve the development and progression of urothelial cancer. In particular, recent investigation, based on striking sex-specific differences in its incidence [1,2], has suggested critical roles of sex hormone receptors, including androgen receptors (AR) (i.e., stimulation) and estrogen receptors (ERs) (i.e., both stimulation and inhibition, potentially dependent on the functional activity of the ERα versus ERβ), in bladder cancer [8,9].

Several glucocorticoids have been clinically used as cytotoxic agents, especially for hematological malignancies [10]. Emerging evidence has also indicated that glucocorticoids inhibit cell growth of solid tumors, such as prostate cancer [10,11,12]. Recently, in contrast to potential unfavorable effects of glucocorticoid therapy, as described above [7], experimental observations have suggested that another steroid hormone receptor, glucocorticoid receptor (GR), functions as a tumor suppressor, in bladder cancer. In this article, we review the available data indicating the involvement of glucocorticoid-mediated GR signaling in urothelial tumor outgrowth and discuss the underlying molecular mechanisms.

## 2. Glucocorticoids, GR, and the Mechanism of Action

Glucocorticoids, a class of steroid hormones, are involved in almost every cellular, molecular, and physiologic event and are major regulators of, for instance, cell growth, energy production, metabolic processes, immune and cardiovascular function, reproduction, recovery from stress, and maintenance of homeostasis. Indeed, glucocorticoids represent one of the most commonly prescribed medications often employed in the treatment of inflammatory and autoimmune disorders.

Glucocorticoids act on gene transcription (positively or negatively), by interacting with the GR, a member of the nuclear receptor superfamily that functions as a ligand-inducible transcription factor. As is the case with other steroid hormone receptors, the classic GR (also known as GRα or GRα-A) undergoes conformational changes upon binding to the glucocorticoids, dissociates from the heat-shock proteins, homodimerizes, and translocates to the nucleus, where it directly interacts with glucocorticoid response elements (GREs) in the promoter of target genes, resulting in the regulation of the GRE-mediated transcription of genes, such as *FK506-binding protein 51* (*FKBP51*) and *glucocorticoid-induced leucine zipper* (*GILZ*) (termed “transactivation”) [13,14]. Glucocorticoid-activated GR dimers also interact with other transcription factors, including activator protein (AP)-1 and nuclear factor (NF)-κB, and indirectly regulate their activity on the target genes (termed “transrepression”) [14,15]. The action of glucocorticoids is often complex and is generally dependent on a balance between transactivation and transrepression of GR. Of note, the therapeutic effects of glucocorticoids are thought to be mainly due to GR transrepression, whereas the adverse effects associated with glucocorticoid therapy are often induced by GR transactivation.

Although the GR is derived from a single gene, multiple GR proteins exist in alternative splicing isoforms (e.g., GRβ, GRγ, GR-A, and GR-P) as well as translational isoforms (e.g., GRα-B, GRα-C1, GRα-C2, GR-C3, GRα-D1, GRα-D2, and GRα-D3) [16]. Of these, GRβ is a major isoform that does not bind to glucocorticoids and exhibits intrinsic GRα-independent transcriptional activity [17]. These functionally distinct receptor subtypes are subject to a variety of post-translational modifications, such as phosphorylation, ubiquitination, sumoylation, and acetylation. The interactions between the isoforms (e.g., dominant negative effects of GRβ on GRα-mediated gene transcription [18]) have also been documented.

## 3. GR Expression in Sex Hormone-Related Cancers

The levels of GR expression have been immunohistochemically assessed in tissue specimens of several types of cancers. Table 1 summarizes the results of the GR immunoreactivity in sex hormone-related tumors, including breast [19,20,21,22], prostate [23], bladder [24], and upper urinary tract [25] cancers. In breast and prostate cancers, the rate of the GR positivity or its overexpression was significantly lower in tumors than in the corresponding non-tumorous tissues, all of which appeared to express the GR. In one of the studies in breast cancer [22], GR expression was shown to be significantly down-regulated in grade 3 tumors, compared with grade 1 or 2 tumors. However, there were no significant differences in the level of GR expression between low and high stage breast cancers [20,22]. In prostate cancer, most of the specimens showed low or no GR expression, which was not significantly associated with the tumor grade (i.e., Gleason score) [23,26]. It is noteworthy that the levels of GR expression have been shown to be augmented, following the treatment with anti-androgenic drugs, in prostate cancer cell lines, xenograft tumors, and surgical specimens [26,27,28,29], implying the involvement of the GR signals in resistance to androgen deprivation therapy. Indeed, physical interactions between the GR and the AR signals have been indicated by demonstrating that AR activation leads to a reduction of the GR expression in prostate cancer cells [30].

The levels of GR expression appear to have been determined in urothelial cancer tissues, only in two studies published by our group [24,25]. We immunohistochemically stained for GR, in our tissue microarrays consisting of a hundred and forty-nine cases of bladder tumor [24] and ninety-nine cases of upper urinary tract tumor [25], as well as their matched normal-appearing urothelial tissues. The positive rates of GR expression were significantly lower in tumors than in the normal-appearing urothelial tissues. In addition, the GR levels were significantly reduced in the high-grade (vs. the low-grade) or the muscle-invasive (vs. the non-muscle-invasive) bladder tumors, while such significant differences were not seen in the upper urinary tract tumors. Moreover, in bladder tumors, GR versus ERα or ERβ were positively or negatively correlated, respectively, while there was no significant correlation between the GR versus the AR.

The prognostic values of GR expression in patients with breast or prostate cancer have not been thoroughly assessed. In a meta-analysis, high levels of GR expression in ER-positive breast cancers were found to be associated with significantly longer relapse-free survival [31]. Similar results, suggesting an association between the GR expression in the ER-positive breast cancer and significantly improved recurrence-free survival, were demonstrated in a more recent study [32]. Conversely, in the ER-negative early-stage breast cancers from those who did or did not undergo adjuvant chemotherapy, high GR expression was associated with worse patient outcomes, compared to no or low GR expression [31,32]. Correspondingly, preclinical studies showed that GR activation diminished the efficacy of chemotherapy and apoptosis in triple negative breast cancer xenograft tumors and that systemic treatment with a GR antagonist could reverse the effects of the anti-cancer agents [33,34]. These findings suggest crosstalk between the GR and ER pathways and the functional role of GR, as a tumor suppressor, only via cooperation with the ER signaling. Similarly, prognostic significance of the GR expression in castration-resistant prostate cancer appears to be dependent on the status of the AR [12].

In patients with bladder cancer, we found that a loss of strong GR expression (i.e., 0/1+ or 0/1+/2+) was also associated with considerably higher risks for the recurrence of non-muscle-invasive tumors and progression of cancer-specific mortality of the muscle-invasive tumors [24]. Multivariate analysis further identified loss or weak positivity of the GR as significant and marginal independent predictors for the recurrence of non-muscle-invasive tumors (hazard ratio = 2.252; 95% confidence interval = 0.936–4.132, *p* = 0.034) and the progression of muscle-invasive tumors (hazard ratio = 3.690; 95% confidence interval = 0.869–15.625, *p* = 0.077), respectively. These immunohistochemical findings in bladder cancer specimens, along with the down-regulation of the GR expression in the urothelial tumors (vs. the non-neoplastic urothelial tissues) and the muscle-invasive tumors (vs. the non-muscle-invasive tumors), strongly suggest a protective/inhibitory role of the GR signals in urothelial tumorigenesis and tumor growth. However, there were no statistically significant associations between the levels of GR expression in the urothelial cancers of the upper urinary tract and the outcomes of the patients [25].

## 4. The Effects of Glucocorticoids on the Growth of Bladder Cancer Cells

As aforementioned, glucocorticoids, such as dexamethasone and prednisone, have often been given to patients with hematological malignancy or castration-resistant prostate cancer [10,12]. Preclinical findings have indeed supported the anti-tumor activities of glucocorticoids in these malignancies, as well as others including cervical, lung, and kidney cancers [11,35,36,37,38]. However, only a few studies have assessed the effects of glucocorticoid treatment on the growth of urothelial cancer cells.

In a study published in 1955 [39], cortisone was shown to induce metastasis in the lung. The T150 transplantable bladder cancer cells derived from a C57BL/6 mouse were subcutaneously injected into the C57BL/6 mice and, after the tumors reached 1 cm in diameter (day 0), the mice received 4–5 injections of 0.5 mg cortisone on alternate days. Lung metastases were grossly identified in thirty-one (77.5%) of the forty mice with cortisone treatment versus eighteen (47.4%) of the thirty-eight control mice at days 14–16.

In two studies, the efficacy of a synthetic glucocorticoid dexamethasone in bladder cancer cell proliferation was assessed. The first study showed no significant effects of dexamethasone on the viability of human bladder tumor cell lines, including 5637, RT-112, EJ28, T24, RT4, and TCC [40]. In the other study [41], however, dexamethasone at 10–1000 nM, significantly induced the growth of GR-positive human bladder cancer lines, TCCSUP, and UMUC3, compared with mock treatment, but not that of their GR knockdown sublines derived following an infection with the GR-short hairpin RNA (shRNA) virus, and the stimulatory effects of dexamethasone on cell proliferation were blocked by RU486 which functions as an GR antagonist. In these studies, dexamethasone was also shown to reduce apoptosis, while inducing cell cycle arrest at the G1 phase (in bladder cancer lines) [41] or S phase (in a cervical cancer line) [40]. Conversely, dexamethasone strongly inhibited the invasion of the TCCSUP/UMUC3 cells and reversed their epithelial-to-mesenchymal transition (EMT) [41]. Inhibition in the migration of T24 human bladder cancer cells was also demonstrated in another study [42]. In accordance with these findings in cell culture systems, dexamethasone slightly induced the growth of the UMUC3 xenografts in mice but strongly inhibited the development of metastasis [41]. Thus, dexamethasone is likely to contrary modulate bladder cancer cell proliferation versus invasion or metastasis.

In a subsequent study [43], the effects of dexamethasone and other nine natural or synthetic glucocorticoids (i.e., betamethasone, budesonide, corticosterone, fludrocortisone acetate, flumethasone, fluticasone propionate, hydrocortisone, prednisone, and triamcinolone) on the growth of bladder cancer cells were compared. Interestingly, corticosterone and prednisone failed to significantly increase the cell viability of both GR-positive and GR-negative TCCSUP/UMUC3 sublines, while the other glucocorticoids tested exhibited stimulatory effects similar to that of dexamethasone, in the GR-positive cells. Nonetheless, corticosterone and prednisone, as well as dexamethasone, similarly inhibited the TCCSUP/UMUC3 cell invasion [43]. As the glucocorticoid potency of corticosterone and prednisone is relatively low yet similar to that of, for example, triamcinolone, it is unlikely to be the main reason for their insignificant effects on the proliferation of the GR-positive bladder cancer cells. Thus, further studies are required to elucidate the underlying mechanisms for the varying degrees of the effects of different glucocorticoids on bladder cancer cell growth.

Prior to the development of effective antiemetic drugs, glucocorticoids were frequently prescribed to patients with advanced bladder cancer, during systemic chemotherapy. Strikingly, previous studies in bladder cancer lines have demonstrated that dexamethasone substantially reduces the cytotoxic effect of an anti-cancer agent cisplatin via inhibition of the cisplatin-induced apoptosis [40,41,43]. However, this phenomenon in bladder cancer cell lines was not seen in the treatment of cisplatin plus corticosterone or prednisone [43]. Glucocorticoids are also used to improve the cachectic conditions in those with the end-stage disease. Thus, clinical use of some glucocorticoids, especially dexamethasone, may rather be harmful to bladder cancer patients with or without undergoing chemotherapy, since they can promote tumor cell proliferation.

## 5. GR and the Related Signals in Bladder Cancer

As seen in dexamethasone treatment, contrary regulation of bladder cancer outgrowth by GR signals might be anticipated. Indeed, dexamethasone was found to induce both transactivation (i.e., induction of the GRE-mediated transcriptional activity and the expression of *FKBP51* and *GILZ* genes) and transrepression (i.e., suppression of the NF-κB and AP-1 transcriptional activities and the expression of NF-κB-regulated genes, such as interleukin (IL)-6, matrix metalloproteinase (MMP)-2, MMP-9, and vascular endothelial growth factor (VEGF)) of GR in the bladder cancer cells [44]. Dexamethasone was also shown to reduce the levels of GR protein expression in bladder cancer cells [41].

In our previous study, comparing the growth of bladder cancer sublines stably expressing control-shRNA versus GR-shRNA, the GR knockdown resulted in significant induction of the migration of the TCCSUP cells [44], suggesting a suppressive role of the GR (i.e., GRα) in urothelial cancer. However, there were no significant differences in cell migration [44], cell invasion [44], and tumor growth in xenograft-bearing mice [41], between the UMUC3-control-shRNA versus the UMUC3-GR-shRNA sublines.

As described above, GRβ, a major isoform of GR, is known to exhibit intrinsic GRα-independent transcriptional activity without glucocorticoid binding [17]. Dexamethasone treatment did not considerably alter the expression levels and localization of GRβ mRNA/protein in human bladder cancer cell lines [42], although it was shown to induce *GRβ* gene expression in mouse embryonic fibroblast cells [45]. In T24 bladder cancer cells stably expressing the GRβ-shRNA, significant inhibition of their migration was observed, compared with a control subline [42]. Additional findings derived from a mutational analysis of the three prime untranslated region in the same study suggested that microRNA-144 might regulate GRβ expression during bladder cancer cell migration [42]. Meanwhile, in addition to immunosuppression [7], the involvement of the GRβ signaling via its activation in bladder cancer development induced by long-term glucocorticoid therapy has also been suggested [46].

In non-urothelial cancer cells (e.g., prostate cancer), modulation of the downstream targets of GR signals by glucocorticoids has been demonstrated [11,37,47,48,49]. Similarly, in bladder cancer cells, up- or down-regulation of the molecules modulated via the glucocorticoid-mediated GR pathway has been shown (Table 2). As a result of inducing transactivation or transrepression of GR by dexamethasone, up-regulation of FKPB51 and GILZ or down-regulation of NF-κB and AP-1 as well as MMP-2, MMP-9, IL-6, and VEGF, respectively, has been documented in bladder cancer cells [41,43,44]. Corticosterone and prednisone were also shown to reduce the expression levels of *MMP-9*, *IL-6*, and *VEGF* genes in bladder cancer cells [43]. In addition, although dexamethasone did not significantly reduce the levels of NF-κB protein expression, it induced the expression of IκB [41], a natural inhibitor of NF-κB, as well as the physical interactions between the GR and the NF-κB [44], in bladder cancer cells.

Again, glucocorticoids have been shown to modulate cell cycle and apoptosis in GR-positive bladder cancer. Additional studies for their associated molecules demonstrated that dexamethasone increased the expression of p21 and p27, and decreased that of the cleaved caspase-3, while there were no significant changes in the expression of cyclins D1/D2/D3 and cyclin-dependent kinase-2/4/6 [41]. Similarly, dexamethasone was found to reverse EMT in the GR-positive bladder cancer cells via an increase in the expression of epithelial markers, such as E-cadherin and β-catenin, and a decrease in that of the mesenchymal markers, such as N-cadherin, Snail, and vimentin, whereas the GR knockdown showed opposite effects on the expression of E-cadherin and β-catenin [41].

GR signals have also been implicated in modulating the function of several transcription factors, including the forkhead box (Fox) protein family. For example, activation of GR was shown to facilitate the reprograming of the chromatin landscape in breast or prostate cancer cells, resulting in the recruitment of FoxA1 [50,51]. Interestingly, FoxA1 was found to be a downstream target of another transcription factor GATA3 [52] that has been used as a critical immunohistochemical marker for not only diagnosing breast cancer in surgical pathology practice but also urothelial differentiation [53]. In addition, dexamethasone treatment in breast cancer cells induces phosphorylation of FoxO3a and reduces its transcriptional activity, resulting in the inhibition of FoxO3a-mediated apoptosis [54]. Nonetheless, the interplay between the GR and Fox proteins has not been documented in urothelial cells. Instead, our preliminary study [55] showed that GR expression in bladder cancer tissue specimens detected by immunohistochemistry was positively and negatively associated with the expression of FoxO1 and its inactive form phospho-FoxO1, respectively. Furthermore, patients with GR(2+/3+) and FoxO1-positive non-muscle-invasive bladder tumor had a significantly lower risk of disease recurrence (*p* = 0.009) and those with GR(2+/3+) and phospho-FoxO1-negative muscle-invasive bladder tumor had a significantly higher risk of disease progression (*p* = 0.012) [55].

## 6. The Potential Interplay of GR with Other Steroid Hormone Receptor Signals in Bladder Cancer Cells

**Figure 1:**
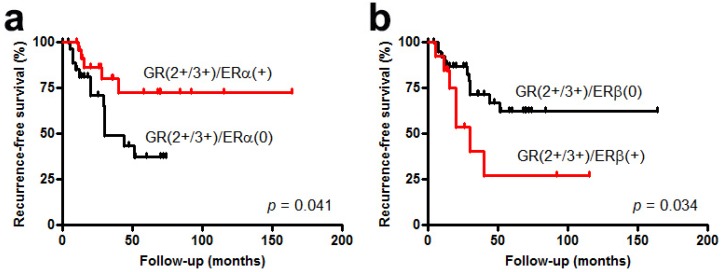
Kaplan-Meier curves for recurrence-free survival in patients with non-muscle-invasive bladder tumor, according to the levels of GR and ERα (**a**: negative (0) twenty-nine cases vs. positive (+) twenty-six cases) or ERβ (**b**: negative (0) forty-two cases vs. positive (+) thirteen cases) expression.

The functional interplay between GR and sex hormone receptors, such as AR and ER, has been suggested. For example, it has been shown that glucocorticoids regulate AR expression in monkey kidney fibroblast COS-1 cells [56], while AR signals inhibit GR gene transcription in human prostate cancer cell lines, androgen-dependent LNCaP and castration-resistant C4-2 [30]. Direct heterodimer formation of the GR and AR via their DNA-binding domains has also been demonstrated [57]. Indeed, glucocorticoids are prescribed particularly in patients with castration-resistant prostate cancer in which elevated GR expression is often seen following androgen deprivation therapy [12,26,30]. Similarly, down-regulation of the GR expression by estrogens and physical interactions between the GR and ERα have been documented in breast cancer cells [58,59,60]. In contrast, the interplay between the GR versus the AR, the ERα, or the ERβ has not been confirmed in urothelial cancer cells. Instead, our immunohistochemistry in bladder cancer specimens identified a positive correlation between the GR and the ERα (*p* = 0.004), as well as a negative correlation between the GR and the ERβ (*p* = 0.007) [24]. In addition, ERα (*p* = 0.041; Figure 1a) or ERβ (*p* = 0.034; Figure 1b) expression in non-muscle-invasive bladder tumors with moderate (2+) to strong (3+) GR expression (*n* = 55) was associated with a significantly lower or higher risk, respectively, of disease recurrence (Ide et al., unpublished data; immunostaining was performed previously in a set of tissue microarray [24,61]). These immunohistochemical findings in surgical specimens imply the involvement of ER signals in the GR-mediated bladder tumor outgrowth. Meanwhile, FoxA1, described above, has been suggested to serve as a pioneer transcription factor that promotes the interactions between the GR and the AR or the ER, primarily in breast cancer cells [50,51,62].

Instead of investigating the potential interplay between the GR and the AR signals in bladder cancer cells, the effects of a unique steroid hormone receptor modulator, compound A [CpdA; 2-(4-acetoxyphenyl)-2-chloro-*N*-methyl-ethylammonium chloride] known to function as not only a GR ligand [63] but also an AR antagonist [64], on their growth have been assessed [44]. CpdA is a synthetic analogue of a hydroxyphenyl aziridine precursor found in the Namibian shrub *Salsola tuberculatiformis* Botschantzev [65]. The anti-tumor property of the CpdA was demonstrated in B-cell lymphoma and multiple myeloma cell lines [66], as well as prostate cancer cell lines [67]. It was also shown that the CpdA could bind to both the GR and the AR at their ligand-binding domains and efficiently competed with dexamethasone (for GR binding) and dihydrotestosterone (for AR binding) [67]. In human bladder cancer cells, we demonstrated that CpdA significantly inhibited cell proliferation of the two GR-positive/AR-positive lines (i.e., TCCSUP, UMUC3) and the two GR-positive/AR-negative lines (i.e., 5637, 647V), as well as the two GR-knockdown/AR-positive sublines (i.e., TCCSUP-GR-shRNA, UMUC3-GR-shRNA) [44]. Similarly, CpdA significantly inhibited the migration and invasion of the GR-positive/AR-positive or the GR-positive/AR-negative cells, as well as the growth of their xenograft tumors in mice. As expected, the efficacy of the CpdA was more significant in the GR-positive/AR-positive cells than in the GR-positive/AR-negative or the GR-negative/AR-positive cells, suggesting its anti-tumor activity via both the GR and the AR pathways. CpdA treatment also resulted in significant decrease in the expression of MMP-2, MMP-9, IL-6, and VEGF in GR-positive/AR-positive and GR-positive/AR-negative sublines, but not in a GR-negative/AR-negative subline. Remarkably, CpdA even more strongly repressed the transcriptional activity of NF-κB and AP-1, compared with dexamethasone, whereas it failed to significantly induce the transcriptional activity or expression of GR, as well as the expression of *FKBP51* and *GILZ* genes. Co-immunoprecipitation further confirmed the induction of the interactions between the GR and NF-κB by not only dexamethasone but also CpdA. It is, thus, likely that the CpdA preferentially induces GR-mediated transrepression, but not transactivation, in bladder cancer cells, suggesting that treatment with the CpdA in vivo may be associated with fewer glucocorticoid-related adverse effects. Of note, we recently showed a functional interplay between NF-κB and AR in bladder cancer cells [68], further indicating the difficulty in deciphering the effects of CpdA on the growth of urothelial tumor cells only through GR signaling.

## 7. Conclusions

The functional role of glucocorticoid-mediated GR signaling has been more extensively investigated in the growth of hematological malignancies, as well as that of solid endocrine tumors, such as breast and prostate cancers, relating to its interplay with ER and AR, respectively. Several glucocorticoids, as promising anti-tumor agents, are also given to patients with hematological malignancy or castration-resistant prostate cancer. In contrast, the efficacy of glucocorticoid treatment has been assessed only in preclinical models of the urothelial cancer. Instead, in patients with urothelial cancer, glucocorticoids have often been given to reduce acute toxicity, particularly hyperemesis, during chemotherapy or radiotherapy, and protect normal tissues against the long-term effects of the genotoxic agents, as well as to improve cachectic conditions. As the down-regulation of GR expression in surgical samples of bladder tumor is strongly associated with the risks for recurrence of a non-muscle-invasive disease, following transurethral tumor resection, and progression of a muscle-invasive disease, following radical cystectomy, GR is thought to function generally as a tumor suppressor for urothelial cancer. However, it is notable that glucocorticoids have been shown to strongly inhibit cell migration/invasion of bladder cancer, but some of them, including dexamethasone, rather promote cell proliferation and reduce apoptosis, especially that induced by cisplatin. Thus, it remains unanswered whether clinical use of glucocorticoids is beneficial or perhaps harmful to patients with urothelial cancer in terms of their unfavorable effects on tumor progression.

Importantly, the underlying molecular mechanisms of how glucocorticoids mediate the progression of bladder cancer through the GR pathway remain poorly understood. In particular, the dual roles of glucocorticoid treatment (i.e., stimulation of cell proliferation vs. the suppression of cell migration/invasion), as well as the differential effects of various glucocorticoids (e.g., stimulation of cell proliferation by dexamethasone vs. no significant stimulation of cell proliferation by corticosterone and prednisone), need to be further investigated. Nonetheless, these findings may be due to the differences in the degree of induction of the GR transactivation versus transrepression by various glucocorticoids. Indeed, the CpdA that selectively induces transrepression has been shown to inhibit not only cell migration/invasion of bladder cancer but also the cell proliferation of even AR-negative bladder cancer lines, primarily via the GR pathway. Treatment with CpdA or other glucocorticoids/selective GR modulators that do not considerably induce GR transactivation may also be eventually beneficial to patients, in order to minimize the associated side effects. Further investigation of glucocorticoids and GR signals, as well as the functional interplay between the GR and the AR or the ERα/ERβ, in urothelial cancer may thus provide novel therapeutic options in patients with bladder cancer.

## Figures and Tables

**Table 1:** GR positivity or overexpression in sex hormone-related cancers in immunohistochemical studies.

Author, Year [Reference]	*N*	Cancer Tissue	Non-Cancer vs. Cancer			Tumor Grade			Tumor Stage			Prognostic Significance (*p* Value)
			Non-Cancer	Cancer	*p* Value	Low	High	*p* Value	Low	High	*p* Value	
Lien, 2006 [19]	347	Breast	100%	2%	<0.001			NA			NA	NA
Conde, 2008 [20]	142	Breast	100%	47%	<0.001			NA	36% (T1-2)	25% (T3-4)	0.706	NA
Buxant, 2010 [21]	133	Breast	100%	40%	<0.001			NA			NA	NA
Abduljabbar, 2015 [22]	999	Breast	NA	62%	NA	71% (G1-2)	52% (G3)	<0.001	61% (stage 1–2)	63% (stage 3)	0.905	NA
Yemelyanov, 2007 [23]	116	Prostate	100%	31%	<0.001	32% (GS6-7)	30% (GS8-10)	1.000				NA
	102	Prostate	100%	13%	<0.001	12% (GS6-7)	15% (GS8-10)	1.000				NA
Ishiguro, 2014 [24]	149	Bladder	96%	87%	0.026	96%	81%	0.011	96% (NMI)	74% (MI)	<0.001	0.025 (RFS of NMI) 0.030 (PFS of MI) 0.067 (CSS of MI)
Kashiwagi, 2015 [25]	99	UUT	84%	63%	0.001	53%	64%	0.563	62% (NMI)	63% (MI)	1.000	NS

UUT: upper urinary tract; NA: not assessed; GS: Gleason score; NMI: non-muscle-invasive; MI: muscle-invasive; RFS: recurrence-free survival; PFS: progression-free survival; CSS: cancer-specific. survival; NS: not significant.

**Table 2:** Targets of the glucocorticoid receptor (GR) signaling modulated by glucocorticoids in bladder cancer cells.

Molecule	Glucocorticoid	Effect	[Reference]
FKBP51	DEX	Up-regulation	[44]
GILZ	DEX	Up-regulation	[44]
NF-κB	DEX	Down-regulation	[44]
	CpdA		
IκB	DEX	Up-regulation	[41,44]
AP-1	DEX	Down-regulation	[44]
	CpdA		
MMP-2	DEX	Down-regulation	[41,43,44]
	CpdA		
MMP-9	DEX	Down-regulation	[41,43,44]
	CORT		
	PRED		
	CpdA		
IL-6	DEX	Down-regulation	[41,43,44]
	CORT		
	PRED		
	CpdA		
VEGF	DEX	Down-regulation	[41,43,44]
	CORT		
	PRED		
	CpdA		
p21	DEX	Up-regulation	[41]
p27	DEX	Up-regulation	[41]
E-cadherin	DEX	Up-regulation	[41]
β-catenin	DEX	Up-regulation	[41]
N-cadherin	DEX	Down-regulation	[41]
Snail	DEX	Down-regulation	[41]
Vimentin	DEX	Down-regulation	[41]

DEX: dexamethasone; CpdA: compound A; CORT: corticosterone; PRED: prednisone.
